# Whole Blood Platelet Aggregation and Release Reaction Testing in Uremic Patients

**DOI:** 10.1155/2013/486290

**Published:** 2013-06-26

**Authors:** Jay Zeck, Jason Schallheim, Susie Q. Lew, Louis DePalma

**Affiliations:** ^1^Department of Pathology, George Washington University Medical Center, 900 23rd Street NW, Washington, DC 20037, USA; ^2^Department of Medicine, George Washington University Medical Center, Washington, DC 20037, USA

## Abstract

*Background*. Platelet function analysis utilizing platelet-rich plasma and optical density based aggregometry fails to identify patients at risk for uremia associated complications. *Methods*. We employed whole blood platelet aggregation analysis based on impedance as well as determination of ATP release from platelet granules detected by a chemiluminescence method. Ten chronic kidney disease (CKD) stage 4 or 5 predialysis patients underwent platelet evaluation. Our study aims to evaluate this platform in this patient population to determine if abnormalities could be detected. *Results*. Analysis revealed normal aggregation and ATP release to collagen, ADP, and high-dose ristocetin. ATP release had a low response to arachidonic acid (0.37 ± 0.26 nmoles, reference range: 0.6–1.4 nmoles). Platelet aggregation to low-dose ristocetin revealed an exaggerated response (20.9 ± 18.7 ohms, reference range: 0–5 ohms). *Conclusions*. Whole blood platelet analysis detected platelet dysfunction which may be associated with bleeding and thrombotic risks in uremia. Diminished ATP release to arachidonic acid (an aspirin-like defect) in uremic patients may result in platelet associated bleeding. An increased aggregation response to low-dose ristocetin (a type IIb von Willebrand disease-like defect) is associated with thrombus formation. This platelet hyperreactivity may be associated with a thrombotic diathesis as seen in some uremic patients.

## 1. Introduction

Bleeding and thrombosis occur frequently in patients with renal insufficiency and uremia [1–3]. Uremic patients scheduled for surgery or invasive procedures would benefit from early identification for bleeding and/or thromboses risks. 

Multiple etiologies contribute to the pathogenesis of uremic bleeding. Proposed mechanisms include platelet storage pool deficiency, abnormal platelet metabolism, endothelial cell changes, increased cyclic adenosine monophosphate (cAMP), abnormal platelet binding to von Willebrand factor (vWF), effects of anemia, and fibrinogen impairment [4–9]. In response to thrombin, secretory mechanisms of ATP and other granule contents are suggested to be diminished in uremia [10, 11]. Mechanisms of thrombotic diathesis include enhanced platelet aggregation, an impaired fibrinolytic system, increased surface exposure of phosphatidylserine and increased fibrinogen, factor 8, and vWF activity [12]. Abnormal platelet function has not been attributable to high concentrations of urea alone, suggesting unique conditions of renal failure associated uremia [13].

Platelet aggregometry has been used to determine the presence of platelet dysfunction in patients with chronic renal failure (CRF) at risk for bleeding or thrombosis. The conventional optical assessment of platelet aggregation by Born is technically demanding, expensive and requires platelet-rich plasma (PRP) [14]. The centrifugation steps needed to make PRP can damage platelets and artificially separate platelets from other in vivo plasma factors [15, 16]. Results involving PRP are conflicting, and platelet responses are variable to different stimuli. Thrombotic diathesis cannot be clearly demonstrated with this assay.

Measuring aggregation by impedance in a newer method [17] uses whole blood and has several advantages with conventional platelet aggregometry [18]. Whole blood samples take less processing time. In addition, since red and white blood cells both affect platelet aggregation, this assay replicates in vivo conditions more closely. The whole blood sample preserves labile factors that also affect platelet functioning in uremia, such as thromboxane A2 and prostacyclin [19]. The addition of chemiluminescence technologies allows for simultaneous monitoring of ATP release to various agonists. Demonstration of ATP release reflects intact signal transduction pathways including cyclooxygenase function and thromboxane A2 generation.

This study determines whether a specific pattern of platelet dysfunction could be seen using whole blood impedance based platelet aggregometry with monitoring of ATP release. We hypothesize that patients with high-stage CKD will have abnormal measurements of whole blood platelet aggregation and ATP secretion given the derangements of uremia on platelets in these patients.

## 2. Materials and Methods

The George Washington University Institutional Review Board approved this study, and the experiment was conducted with the understanding and the consent of the human subjects. Nephrologists identified eligible subjects from the clinic at the George Washington University, Medical Faculty Associates (MFA) or the George Washington University Hospital (GWUH). The inclusion criteria for consented subjects were CKD stage 5 not on dialysis (estimated glomerular filtration rate (eGFR) < 15 mL/min per 1.73 m^2^) or CKD Stage 4 (GFR = 15–29 mL/min) as defined by the National Kidney Foundation practice guidelines [20]. Subjects' blood samples were obtained prior to the initiation of dialysis, thus avoiding any confounding effects of partially treated uremia (residual syndrome). Exclusion criteria included patients who had taken any medications within the preceding two weeks that may affect platelet function, such as aspirin, other nonsteroidal inflammatory drugs, thienopyridines, glycoprotein IIb/IIIa receptor antagonists, dipyridamole, and penicillins [21].

The MFA and/or GWUH medical record provided the subjects' demographics, medical history, medication list, eGFR, serum creatinine, urinalysis, blood urea nitrogen (BUN), hemoglobin, hematocrit, and platelet count. 

A single venipuncture obtained approximately ten milliliters of blood from each enrolled subject using standard protocol. The venipuncture was performed by an experienced phlebotomist either in the phlebotomy lab sponsored by the MFA or at the subject's bedside at GWUH. Samples were collected in five to seven plastic tubes containing sodium citrate 3.2%. Clot-free samples remained at room temperature and were transported directly to the GWUH's clinical pathology laboratory and analyzed within 2 hours from collection on a Chrono-Log Model 700 Whole Blood/Optical Lumi-Aggregometer using Aggro/Link8 windows software (Chrono-Log Corporation, Havertown, PA 19083, USA). The aggregometer measured both platelet aggregation and ATP release. The laboratory used standardized protocols and instructions provided by the manufacturer.

The aggregometer measured platelet aggregation as a function of percent electrical impedance (ohms). Briefly, the process consists of immersing an electrode in the blood sample onto which platelets attach uniformly in a monolayer. Once an agonist is added, platelet clumps form on the electrode and thus affect the impedance across it in a measureable way. The platelet agonist reagents (Chrono-Log Corporation, Havertown, PA, USA) included collagen (2 *μ*g/mL and 5 *μ*g/mL), arachidonic acid (AA, 0.5 mM), adenosine diphosphate (ADP, 10 *μ*M), and ristocetin (0.25 mg/mL and 1.25 mg/mL). 

During the same analysis, samples were measured for the ATP release reaction from platelet dense granules. A high-gain photomultiplier tube recorded the binding of platelet secreted ATP to an added firefly luciferin luciferase (chromolume, Chrono-Log Corporation). This emitted light was thus proportional to the secreted ATP (nmoles) following the addition of platelet agonist reagents (Chrono-log Corporation, Havertown, PA 19083, USA), including thrombin (1 unit), collagen, AA, and ADP. The latter three agonists were at the same concentration as that used for the platelet aggregation phase of testing.

Results were correlated to the clinical data and compared to reference ranges. These reference ranges were established in house by the GWUH Pathology Laboratory [16, 22] and obtained by evaluating twenty healthy volunteers with no clinical or laboratory evidence of renal disease. Each reference range represents the interval that is 95% of values from control groups of healthy volunteers. Data on each study participant for each platelet agonist was expressed as a mean ± 1 standard deviation. 

Informed consent for all participants in this study was obtained, and the study adhered to the provisions of the Declaration of Helsinki.

## 3. Results

A total of ten subjects meeting inclusion criteria were analyzed. 

Table 1 summarizes subject characteristics. The subjects' ages range from 39 to 79 years old.

**Figure 1:**
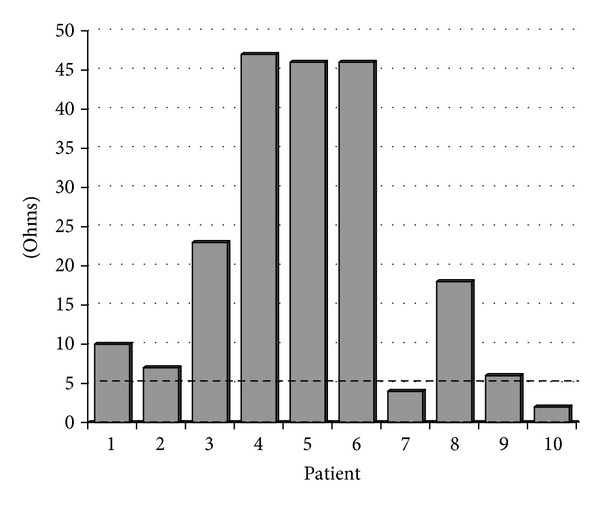
Graphical results of platelet aggregometry to ristocetin 0.25 mg/mL. Dashed line represents the bound of normal reference range (0–5 ohms). Eight of ten patients demonstrate aggregation higher than normal to low-dose ristocetin.

Tables 2 and 3 describe the results of the study. As seen in Table 2, the mean values for platelet aggregation responses to collagen (2 *μ*g/mL and 5 *μ*g/mL), AA, ADP, and high dose ristocetin (1.25 mg/mL) fall within the normal reference range. As noted in Table 2, and illustrated in Figure 1, platelet aggregation to low-dose ristocetin (0.25 mg/mL) reached approximately four times the limit of the upper range (mean of 20.9 ohms versus 5 ohms for the upper limit of normal).

**Figure 2:**
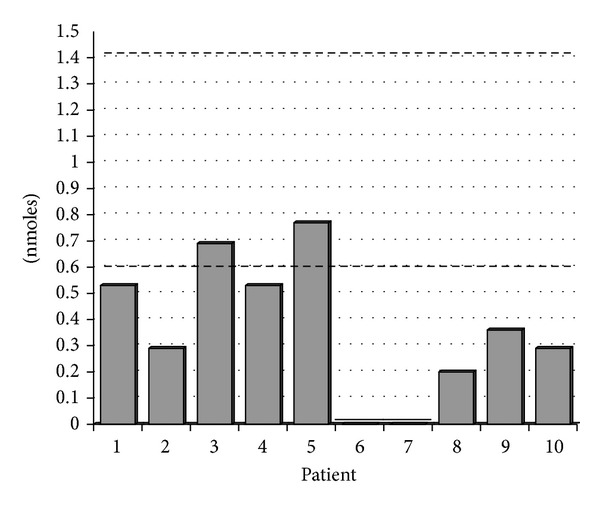
Graphical results of platelet ATP release to arachidonic acid 0.5 mM. Dashed lines represent bounds of normal reference range (0.6–1.4 nmoles). Eight of ten patients demonstrate lower than normal ATP release to the agonist arachidonic acid.

As seen in Table 3, the mean values for ATP release to thrombin, low- and high-dose collagen, and ADP remained in the normal reference range. As noted in Table 3 and illustrated in Figure 2, platelet ATP release to the agonist AA was below the lower limit of the normal reference range (0.37 nmoles versus 0.6 nmoles for the lower limit of the normal reference range).

## 4. Discussion

Our lab has recently acquired whole blood platelet aggregation technology that can simultaneously test both platelet aggregation and the ATP release reaction. We evaluated both of these parameters in uremic patients with CKD stage 4 or 5 just prior to each patient starting hemodialysis. The current literature supports platelet dysfunction in uremia being a cause for bleeding and/or thrombosis, but the nature of the dysfunction has yet to be clearly elucidated. This study further contributes to the understanding of the effects of CKD associated uremia on platelet function. 

Use of platelet-rich plasma (PRP) traditionally helped to describe bleeding tendencies in uremia, but has limitations in illustrating thrombotic diathesis. This study avoids PRP as the aggregation substrate because of inconsistencies with methodology and reproducibility [17, 18, 23–25]. Whole blood from simple venipuncture provides a quick, relatively inexpensive means of attaining platelet aggregation and ATP secretion data from a sample that relates more closely to in vivo conditions. 

Some authors suggest whole blood platelet aggregation in uremia to be normal as compared to controls [24]. Other authors have found aggregation to be decreased [7] or increased [10, 26]. The preponderance of the conflicting results in the literature comes from studies that have examined uremic platelet aggregation using the less accurate PRP based optical methods. Furthermore, the chronic kidney disease subjects evaluated in these studies had already started on chronic dialysis. Our study examines whole blood of patients prior to the initiation of dialysis and thus avoids the complexities of partially treated uremia in dialyzed patients [5, 27]. Even so, the whole blood impedance based results of our study parallel the literature with no specific trends in aggregation results for low-dose collagen (2 *μ*g/mL), high-dose collagen (5 *μ*g/mL), or ADP. It should be noted that no one has reported a correlation between the degree of uremia and platelet aggregation impairment [25]. We also confirm no such correlation in our study, specifically in CKD stage 4 and stage 5 patients. 

To our knowledge, no previous literature systematically studied whole blood platelet aggregation in response to AA or low-dose ristocetin in uremic patients. Platelet aggregation responses to AA in our study showed varying results: 40% of values, decreased, 30% had normal measurements and 30% were increased. Some authors have theorized that uremic toxins only cause defective aggregation in the high shear forces of the vascular flow system and not within the artificial environment of the aggregometer [4]. Others have suggested that bleeding tendencies may not relate to aggregation at all but more so to platelet adhesion and/or secretion [24]. Defects in cyclooxygenase can be investigated by addition of this enzyme's substrate, AA. Eighty percent of the uremic patients in our study showed low levels of ATP release in response to the AA agonist. Free arachidonic acid is converted by cyclooxygenase to prostaglandins G2 and H2. These prostaglandins are then converted by thromboxane synthase to thromboxane A2. This latter molecule promotes additional platelet activation and platelet aggregation with dense granule release of ATP. Our results suggest that there is inhibition of this cyclooxygenase pathway. Indeed the findings simulate an aspirin-like effect. The literature has suggested defective prostaglandin synthesis in uremic platelets involving the cyclooxygenase pathway as implied by decreased generation of thromboxane A2 in response to addition of AA, ADP, thrombin, and collagen [19, 28]. Curiously, thromboxane A2 levels normalize or even increase in patients after initiating dialysis [29, 30]. Our study provides evidence that uremia diminishes cyclooxygenase activity. 

Defects of ATP release in uremic platelet dysfunction parallel observations in inherited platelet function disorders, even with normal aggregation results [31]. Pai et al. found more commonly reduced platelet ATP release in response to one or more agonists in patients with bleeding disorders as compared to healthy control subjects [31]. Of note, relatively mild platelet abnormalities alter in vitro aggregation studies, which may not be prognostic of actual bleeding risk [32, 33]. Our study documents an aspirin-like defect as a likely factor contributing to a bleeding diathesis seen in CKD patients. This finding was evident based on ATP release to AA. In fact, impairment of the cyclooxygenase pathway would not have been detected in 60% of the patients if it is based only on platelet aggregation responses to AA.

Interestingly, 80% of the patients in our study had increased platelet aggregation to low-dose ristocetin (0.25 mg/mL). This differs from reported whole blood based data from Ho et al., where the minority of the uremic patients in their study had impaired ristocetin impedance [25]. However, this study investigated patients already on dialysis and many patients concomitantly on antiplatelet therapy. 

Uremic patients may have a simultaneous risk factor for hypercoagulability as suggested by an exaggerated platelet aggregation response to low-dose ristocetin. This latter finding resembles that seen in type IIb von Willebrand disease and the platelet type of von Willebrand disease. These patients may also have a predisposition to thrombus formation. Testing with high-dose ristocetin cannot detect patients with a potential thrombotic diathesis; therefore, low-dose ristocetin should be employed in patients at risk for hypercoagulability as in uremia.

Whole blood platelet aggregation analysis with measurement of ATP release can be used to identify uremic patients who may be at risk for platelet based bleeding or thrombotic complications. Additional studies should focus on elucidating the mechanisms of our study's findings in an attempt to better determine both a diagnostic approach and the appropriate selection of therapeutic agents in uremic patients with either bleeding and/or hypercoagulability. 

## Figures and Tables

**Table 1:** Characteristics of study subjects.

Patient	Age	Gender	CKD	CR	BUN	eGFR	HGB	HCT	PLT
1	61	M	4	3.36	55	23	9.8	30	239
2	56	M	4	3.44	58	22	9.9	30	195
3	54	F	5	10.1	77	5	8.7	26.9	275
4	39	F	5	8.61	75	6	10.5	34.3	444
5	79	M	4	3.65	46	20	10.1	30.4	330
6	66	F	5	12.15	92	3	5.4	16.6	205
7	69	F	5	8.3	52	4	9.2	26.8	203
8	52	M	5	6.04	32	11	13.2	39.9	274
9	61	F	5	4.15	51	13	8.7	27.1	269
10	46	F	5	15	94	3	9.4	27.9	241

Mean ± Std dev	58 ± 11.6			7.48 ± 4.06	63 ± 20	11 ± 8	9.5 ± 1.93	29.0 ± 5.95	268 ± 74.5

CKD: chronic kidney disease stage, CR: serum creatinine; BUN: blood urea nitrogen, eGFR: estimated glomerular filtration rate, HGB: hemoglobin, HCT: hematocrit, and PLT: platelets. Reference ranges: CR: 0.76–1.27 mg/dL; BUN: 5–26 mg/dL; eGFR > 59 mL/min/1.73; HGB: 12.5–17.0 g/dL, HCT: 36.0–50.0%, and PLT: 140–415 × 10^3^/*μ*L.

**Table 2:** Results of platelet aggregometry (values expressed in ohms).

Patient	Collagen 2 *μ*g/mL	Collagen 5 *μ*g/mL	AA 0.5 mM	ADP 10 *μ*M	Ristocetin 0.25 mg/mL	Ristocetin 1.25 mg/mL
1	0	28	0	24	10	46
2	28	26	14	22	7	46
3	27	26	15	22	23	44
4	19	41	34	28	47	47
5	30	33	30	26	46	45
6	24	30	21	19	46	30
7	6	12	0	21	4	8
8	10	15	0	29	18	28
9	7	13	3	28	6	28
10	12	19	6	5	2	25

Mean ± Std dev	16.3 ± 10.7	24.3 ± 9.43	12.3 ± 12.7	22.4 ± 6.98	20.9 ± 18.7	34.7 ± 13.0
Ref	15–27	15–31	5-17	6–24	0–5	>5

AA: arachidonic acid and ADP: adenosine diphosphate.

**Table 3:** Results of platelet ATP release reaction (values expressed in nmoles).

Patient	Thrombin 1 U	Collagen 2 *μ*g/mL	Collagen 5 *μ*g/mL	AA 0.5 mM	ADP 10 μM
1	0.89	1.06	2.05	0.53	0.18
2	0.9	0.79	0.88	0.29	0.16
3	3.63	3.06	3.74	0.69	1.49
4	1.21	1.52	1.24	0.53	0.37
5	1.55	1.8	1.56	0.77	0.4
6	3.5	0.22	0.18	0	0.7
7	0.82	0.41	0.73	0	3
8	1.15	0.52	0.83	0.2	0.64
9	0.9	0.62	0.63	0.36	1.47
10	0.93	0.26	0.67	0.29	0.14

Mean ± Std dev	1.55 ± 1.09	1.03 ± 0.89	1.25 ± 1.02	0.37 ± 0.26	0.86 ± 0.90
Ref	>0.5	0.5–1.7	0.9–1.7	0.6–1.4	0.38–1.71

AA: arachidonic acid and ADP: adenosine diphosphate.
